# Comparative evaluation of bioactive alkasite-based material in different application modes: a 1-year randomized controlled clinical trial

**DOI:** 10.1007/s10266-025-01095-4

**Published:** 2025-04-15

**Authors:** Nada A. El-Salamouny, Waleed A. Elmahy, Ahmed A. Holiel

**Affiliations:** Conservative Dentistry Department, Faculty of Dentistry, Alexandria University, Alexandria, Egypt

**Keywords:** Cention N, Alkasite based, Bulk fill, FDI criteria, Clinical performance

## Abstract

This study evaluated the one-year clinical performance of Cention N, a new alkasite bioactive restorative material, compared to a conventional bulk-fill composite resin in 12 patients aged 18–45 years with class I cavities. The patients were randomly assigned to three groups: Group I (Cention N without adhesive), Group II (Cention N with adhesive), and Group III (Filtek Bulk Fill composite resin). The restorations were monitored at baseline, three, six, and twelve months using the FDI criteria, assessing marginal staining, recurrent caries, and postoperative sensitivity. Statistical analysis was performed using the Monte Carlo correction, chi-square test, and Friedman’s test (*p* ≤ 0.05). The results showed clinically acceptable FDI scores for all restorations at each time interval with no significant differences between the groups (*p* ≥ 0.05). However, Cention N without adhesive demonstrated slightly inferior outcomes in postoperative sensitivity and marginal staining at the six- and twelve-month intervals. Overall, both materials performed similarly in terms of clinical performance within the first year, though Cention N without adhesive showed marginally lower, but still acceptable, results.

**Trial registration:** ClinicalTrials.gov NCT06273410: 13/10/2022.

## Introduction

Currently, composite resin is the most prevalent direct restorative material. Since their introduction, composites have undergone significant advancements, leading to substantial improvements in clinical performance [1]. These developments include modifications in composition, light-curing protocols, and restorative techniques, all aimed at enhancing material durability [2]. Advancements in material science have led to the bulk-fill era, revolutionizing composite restorations. Bulk-fill composites allow for increment thicknesses of 4–5 mm, offering greater curing depth, reduced polymerization shrinkage, and improved wear resistance compared to conventional composites [3]. Additionally, bulk-fill materials provide several clinical advantages, including shorter treatment times, reduced air void entrapment, and enhanced restoration quality [4, 5].

However, it is pertinent to acknowledge that a marginal gap is likely to occur between the cured material and the cavity walls in any material that sets through a polymerization reaction. This gap facilitates microleakage of bacteria at the margin of restoration, leading to post-operative hypersensitivity, staining and secondary caries [6]. Therefore, the optimum solution is to create materials containing reactive fillers to shield the tooth from secondary caries. These materials are identified as "bioactive restoratives" [7, 8]. Bioactive materials release various remineralizing ions into the oral cavity and at the tooth restoration interface, increasing the longevity of restorations and reducing the risk of recurrent caries [9, 10]. Additionally, they can react to changes in pH, neutralizing the oral environment and consequently eradicating bacteria and promoting remineralization. Recently, new dental materials were developed to address the polymerization shrinkage of resin-based materials and the mechanical drawbacks of GICs [11].

Among these materials are dual-cured, bioactive alkasite restorative materials. This new category incorporates a distinctive iso filler that minimizes polymerization shrinkage by functioning like a spring, gradually expanding as the interactions between fillers intensify during the polymerization phase [12]. Cention N, a newly developed tooth-colored filling material, is intended for bulk application [12, 13] and is basically a composite resin subgroup. This alkasite restorative material has great flexural strength and is indicated for class I, II, or V permanent restorations as well as deciduous teeth. It was claimed to be an amalgam substitute, that can be applied with or without adhesive [14]. The powder contains patented fillers which release "acid-neutralizing" ions and iso fillers which decrease polymerization shrinkage, while the monomer in the liquid improves the material's flowability and allows it to adapt to the smear layer [15, 16].

Class I cavities, characterized by their high configuration factor (C-factor), pose significant challenges due to increased polymerization shrinkage and microleakage. Despite advancements in dental materials, complications such as postoperative hypersensitivity and marginal discoloration persist, highlighting the need for continued innovation in material composition and adhesive techniques. Cention N has demonstrated promising in vitro results, including reduced microleakage and effective ion release [17, 18]. However, clinical evidence on its in vivo performance, particularly in high-stress Class I restorations, remains limited. Additionally, there is ongoing debate over whether adhesive application is necessary for optimal clinical outcomes [19–21]. Most existing research focuses on in vitro parameters [17, 18, 22, 23], which, while valuable, fail to fully replicate the complexities of the oral environment, such as dynamic pH changes, masticatory forces, and patient-specific variables. Thus, well-designed clinical trials are crucial to assess the long-term performance of bioactive materials like Cention N under real-world conditions. This randomized controlled clinical trial aimed to bridge this gap by evaluating the clinical performance of Cention N, with and without adhesive, compared to Filtek Bulk Fill in Class I restorations over a one-year period.

## Methods

### Subjects, study design and setting

**Fig. 1:**
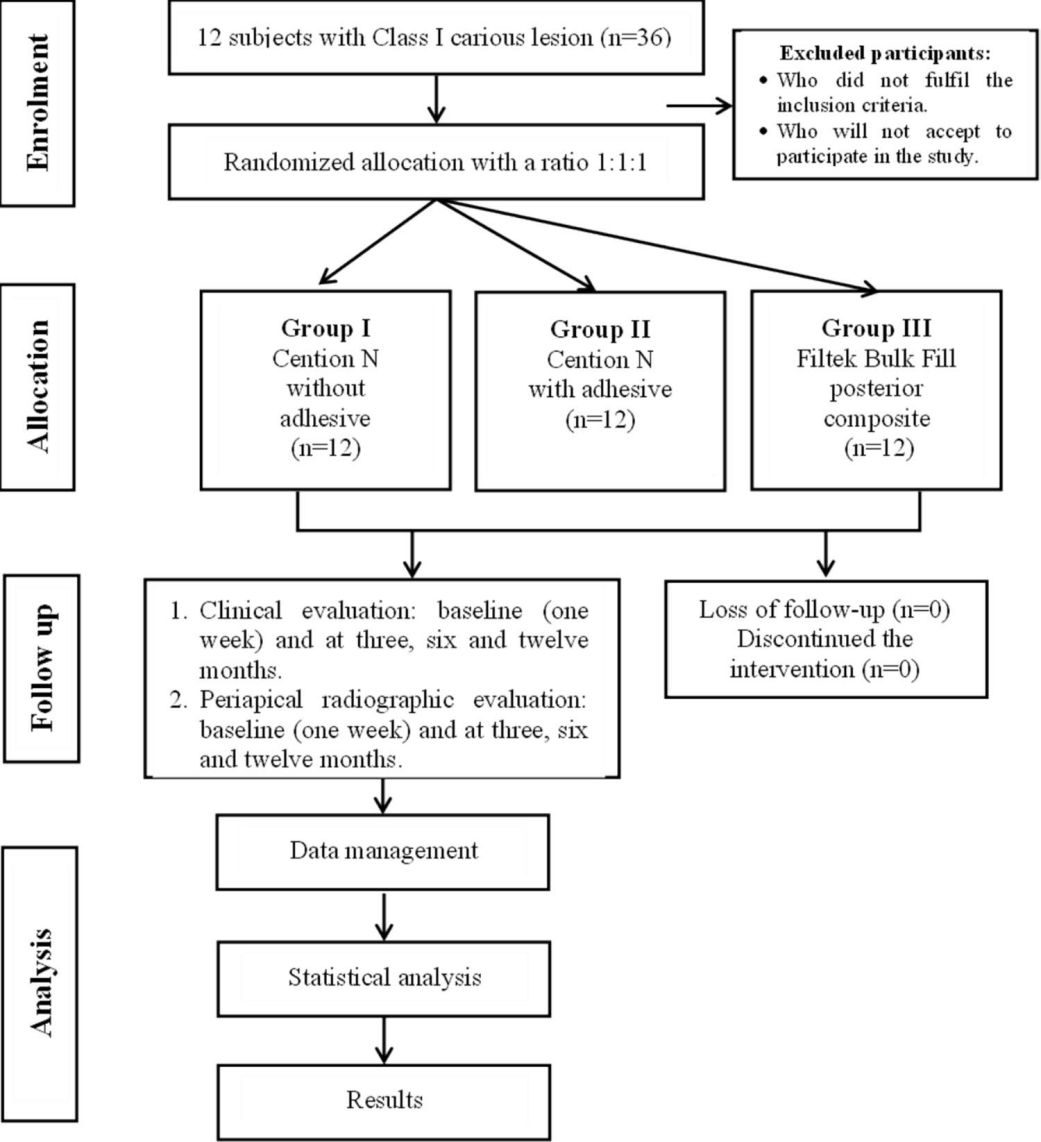
Consort chart of the clinical study

This was a controlled, double-blind randomized clinical trial using three research groups with equally distributed allocations conforming to the guidelines of the Consolidated Standards of Reporting Trials (CONSORT) [24] (Fig. 1). Our current study was ethically approved by the Alexandria University Committee of Research Scientific Unit. Additionally, the current study was registered at ClinicalTrials.gov as NCT06273410. Participants were briefed on the protocols and signed a consent form before being included. Between October 2022 and November 2023, the clinical procedures and evaluations were performed.

### Inclusion and exclusion criteria

In the current study, 12 patients (18–45 years old) were included and treated in the conservative department clinics, Faculty of Dentistry, Alexandria University, at Alexandria, Egypt. Patients with 3 occlusal pit and fissure caries, displaying good oral hygiene and have occlusal contact with opposing teeth were included [25]. However, teeth exhibiting any pathologic pulpal disease, surface loss due to wear, or unsuitable for isolation by rubber dam were rejected from this trial. Teeth that have undergone previous restorations or any defective restoration that is opposing or adjacent to the involved tooth were further eliminated [25].

### Sample size calculations

For power analysis, G*Power 3.1.9.2 software (Franz Faul, Universität Kiel, Germany) was used. Adopting an 80% power (*b* = 0.20) and a 5% significance level (α error allowed = 0.05) to identify a standardized effect size in FDI (primary outcome) of 0.569 based on previous research [26]. Ten cavities per group were determined to be the minimal sample size required (number of groups = 3; total sample size = 30 cavities) [27]. The sample size was raised to twelve cavities per group (number of groups = 3) (total sample size = thirty-six cavities) in anticipation of a 10% dropout rate [28].

### Randomization and allocation concealment

Distribution concealment was ensured by the means of opaque sealed envelopes that were sequentially numbered and sealed until the volunteers’ participation date. The envelope was opened, and the allotted treatment was administered to each tooth after the patient gave permission to be part of the trial. The envelopes were prepared by a faculty member who did not participate in any element of the clinical investigation. When the intervention was administered, the operator was not blinded to the treatment groups. However, neither the evaluators nor the participants were aware of the allocation assignment.

### Intervention

#### Preoperative assessment

**Fig. 2:**
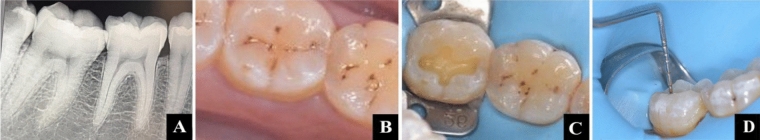
**A** perioperative radiograph, **B** preoperative intraoral photograph showing pit and fissure caries, **C** class I cavity preparation, **D** measuring cavity depth

Medical and dental history were documented for all participants in this study [25, 29]. A dental syringe held 2cm from the tooth surface was used to administer air for ten seconds to assess preoperative sensitivity. Cotton rolls were placed over the neighboring teeth to protect them from the air effect [30]. Prior to commencing the restorative application, all groups underwent the following procedures: All participants received instructions on proper oral hygiene one week preceding the intervention [31]. A low-speed handpiece with pumice slurry in a rubber cup was used to clean all the teeth, followed by rinsing and drying to eradicate any residual biofilm [32]. Preoperative intraoral digital photographs and radiographs were also attained Fig. 2A, B.

#### Clinical procedure

The selected teeth were anaesthetized, and cavity preparation was performed by a highspeed handpiece using a 245-carbide bur under an ample amount of water. The bur was replaced after 5 cavities. The carious lesion's dimensions dictated the cavity's geometry, the cavity's depth of 1.251.5 mm [16, 26] was further determined using a periodontal probe to ensure uniformity in cavity size Fig. 2C, D [29]. Pulpal walls were made flat with rounded line and point angles. Following preparation, rubber dam was utilized for isolating teeth to achieve complete moisture control. Cavities were then cleaned, dried, and inspected for debris. The teeth were allocated at random into three groups: Group (I) Cention N without adhesive, Group (II) Cention N with adhesive and Group (III) Filtek Bulk Fill posterior composite.

**Fig. 3:**
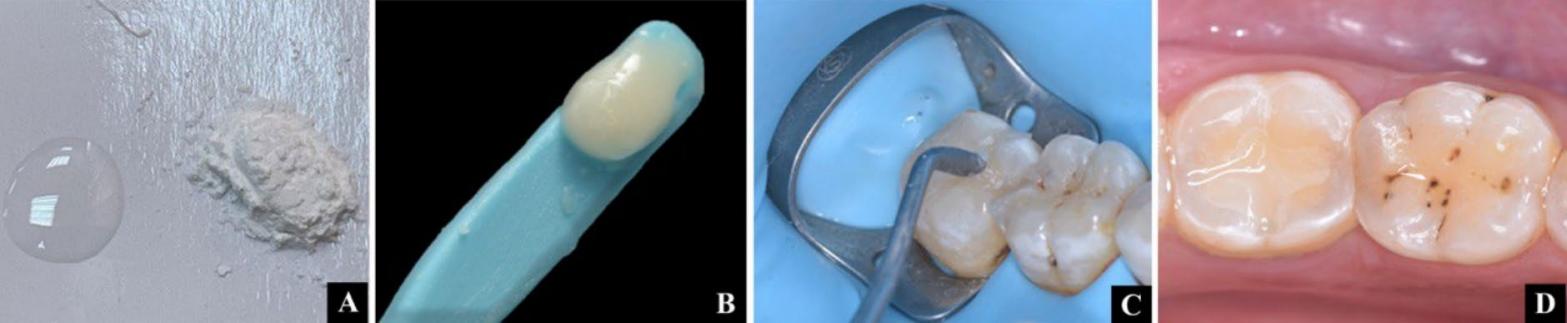
Complete treatment sequence of Group I (Cention N without adhesive) **A**, **B** Mixing of powder and liquid, **C** Application of material, **D** Immediate post operative photograph

*Group I:* Cention N was manually mixed using the standard 4.6:1 powder-to-liquid ratio (one scoop of powder for every drop of liquid) following the manufacturer’s instructions. The powder and liquid were dispensed onto the mixing pad and combined until a uniform consistency was reached, using a plastic spatula (4560 s) Fig. 3A, B. From the onset of the mixing, the working period lasted three minutes. The application of Cention N (shade A2) was done in bulk, with careful adaptation and condensing [16, 25] followed by removal of any occlusal excess, preliminary surface contouring then a 20 s light cure using Elipar Deep Cure LED curing light (3M ESPE, St Paul, USA) with an output irradiance of 1470 mW/cm^2^, designed for the polymerization of light-curing dental materials Fig. 3C, D.

**Fig. 4:**
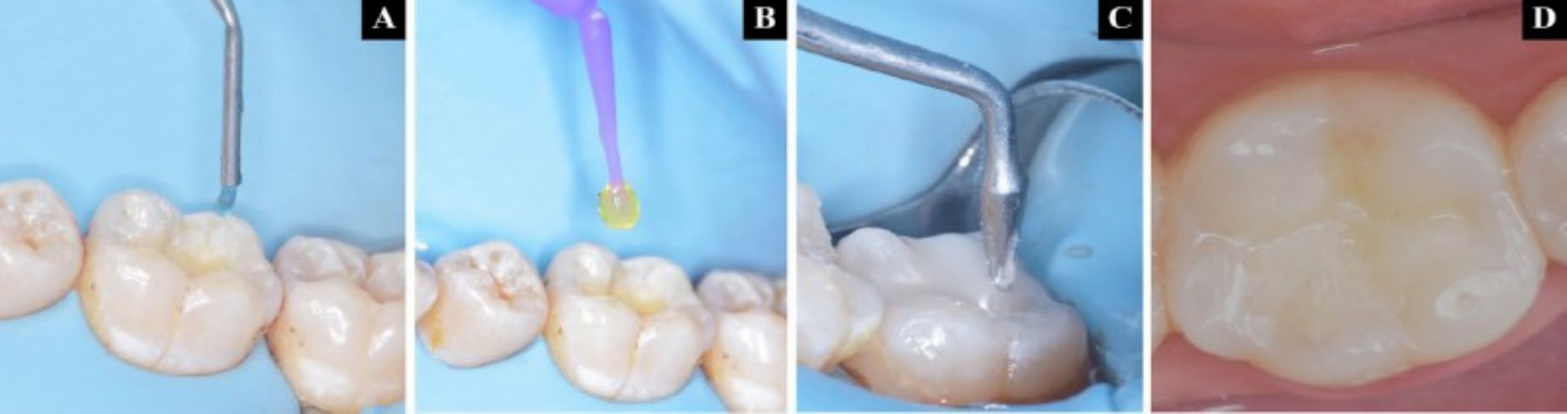
Complete treatment sequence of Group II (Cention N with adhesive) **A**, **B** Selective etching and bonding, **C** Application of material, **D** Immediate post operative photograph

*Group II:* Selective enamel etching of the prepared cavity was carried out for 15 s with N-etch (Ivoclar Vivadent). Thorough rinsing of the etchant with a water jet followed by gentle drying with an air jet [16, 25] Using a disposable applicator, the dentin and enamel surfaces were covered with a substantial coating of Tetric N Bond universal (Ivoclar Vivadent). The adhesive was carefully brushed into the dentin for at least 10 s. To ensure that the enamel and dentin are completely covered by the adhesive without pooling, a gentle air stream was utilized to remove the surplus material followed by a 10 s light cure [16]. Cention N was then manipulated and condensed into the cavity followed by a 20 s light cure Fig. 4A–D.

**Fig. 5:**
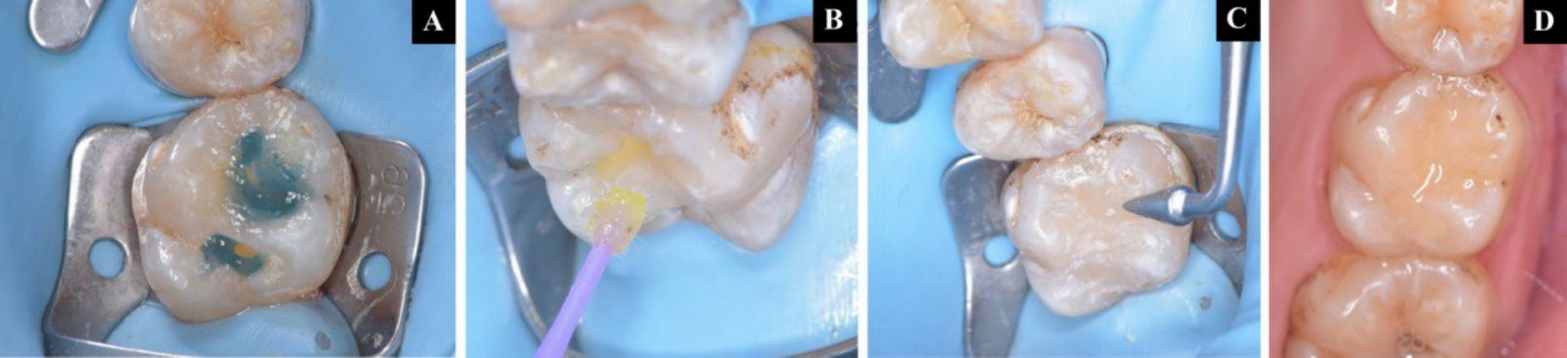
Complete treatment sequence of Group III (Filtek Bulk Fill composite) **A**, **B** Selective etching and bonding, **C** Application of material, **D** Immediate post operative photograph

*Group III:* Selective enamel etching was performed as previously described. Filtek Bulk Fill Posterior Restorative, (3M ESPE, St. Paul, USA) was bulk-filled inside the cavity and then anatomically carved followed by a 20 s light cure [26] Fig. 5A–D.

Following rubber dam removal, occlusion was analyzed for all groups by articulating paper, and fine and ultra-fine diamond burs and the Sof-Lex system (3M ESPE, St Paul, USA) were used to finish and polish the restorations. All restorative treatments were performed by a single skilled operator in the conservative dentistry department, and an immediate postoperative periapical radiograph using a paralleling cone technique was taken.

### Calibration and data collection

Before participating in the study, a collaborative evaluation of twenty direct composite restorations from individuals not involved in the study was used to calibrate the evaluators [33]. Intra and inter-examiner agreement was assessed using Intra Class Correlation Coefficient (ICC) that indicated a good agreement and ranged from 0.76 – 0.83. Furthermore, a paralleling cone approach using an EndoRay aiming device was employed to take a reproducible, standardized periapical digital radiograph. All patients had uniform exposure parameters using a Genoray portable x-ray system with a tube current of 2 mA, tube voltage of 60 kVp, and processed through a DIGIRAY FireCR Dental Reader. Using OnDemand3D, App 1.0.9.3223software, the x-rays were viewed and examined.

### Evaluation of restorations

**Fig. 6:**
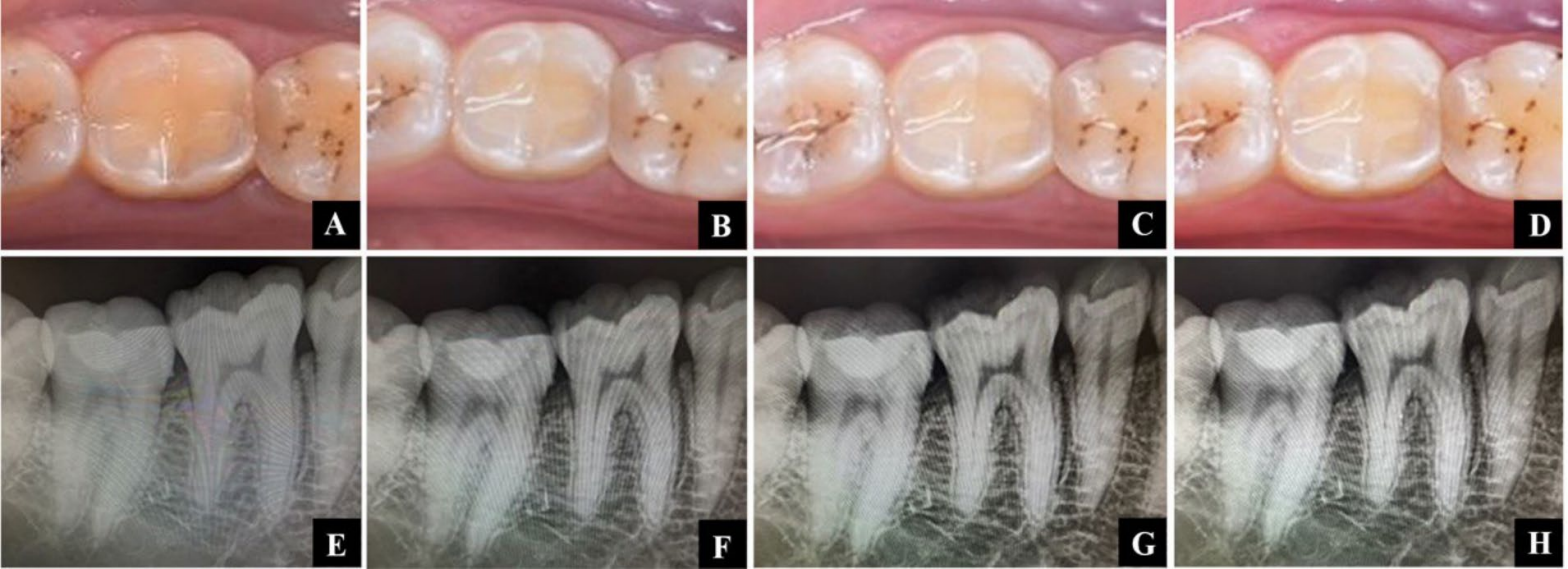
Follow-up sequence of Group I (Cention N without adhesive) **A**-**D** 1week,3-, 6-, and 12- month follow-up images **E**–**H** 1week,3-, 6-, and 12- month follow-up radiographs

**Fig. 7:**
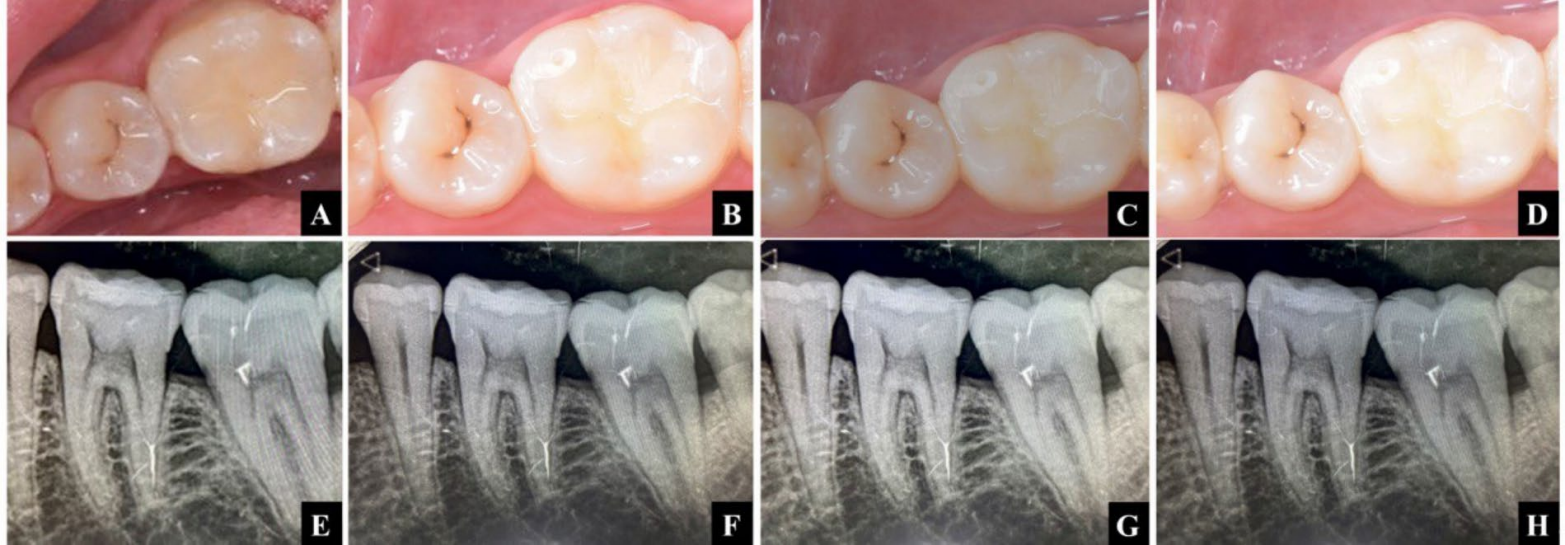
Follow-up sequence of Group II (Cention N with adhesive) **A**-**D** 1 week, 3-, 6-, and 12-month follow-up images **E**–**H** 1 week, 3-, 6-, and 12-month follow-up radiographs

**Fig. 8:**
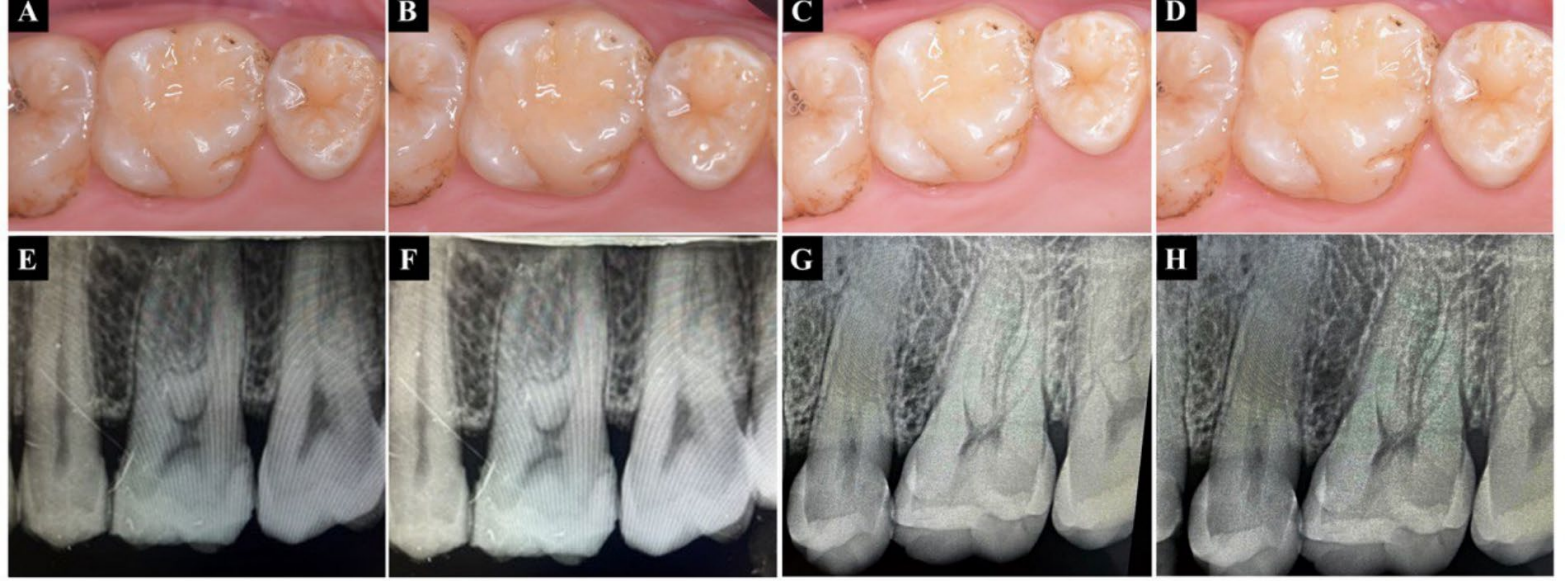
Follow-up sequence of Group III (Filtek Bulk Fill composite) **A**-**D** 1 week, 3-, 6-, and 12-month follow-up images **E**–**H** 1 week,3-, 6-, and 12-month follow-up radiographs

Restoration assessments were carried out at baseline (one week) followed by three, six and twelve months utilizing FDI criteria (Table 1). The clinical evaluation was conducted by two qualified and experienced evaluators who were not involved in the operative process and as a result were blinded to the treatment allocation. In the event of examiner disagreement, it was resolved immediately through joint re-examination and discussion while the patient remained present. Postoperative sensitivity, marginal staining and recurrent caries were the criteria selected for evaluation. Visual and tactile examinations aided with postoperative intraoral photos, and radiographs obtained at each recall appointment were used for conducting the evaluation process Figs. 6, 7, 8. World Dental Federation's assessment criteria for dental restorations are based on a scale of 1 to 5 with the following scores: (1) clinically excellent/very good; (2) clinically good; (3) clinically satisfactory; (small flaws, no unacceptable effects, but not adjustable with/or damage to the tooth); (4) clinically unsatisfactory but repairable; and (5) clinically poor/irreparable that requires necessary replacement. Consequently, scores 1, 2, and 3 are regarded as clinically successful, while scores 4 and 5 are deemed as clinically unsuccessful [34].

**Table 1:** FDI criteria used for clinical evaluation

	Esthetic properties	Biological properties	
	1.Marginal staining	2.Postoperative sensitivity	3.Recurrence of caries
1. Clinically excellent/very good	1.1 No marginal staining	2.1 No hypersensitivity, normal vitality	3.1 No secondary or primary caries
2.Clinically good (after polishing very good)	1.2 Minor marginal staining, easily removable by polishing	2 .2 Minor hypersensitivity for a limited period of time, normal vitality	3.2 Small and localized demineralization No operative treatment required
3. Clinically sufficient/ satisfactory (minor shortcomings, no unacceptable effects but not adjustable without damage to the tooth)	1.3 Moderate marginal staining, not esthetically unacceptable	2.3.1 Moderate hypersensitivity 2.3.2 Delayed/mild sensitivity; no subjective complaints, no treatment needed	3.3 larger areas of demineralization, but only preventative measures necessary (dentin not exposed)
4.Clinically unsatisfactory (but reparable)	1.4 Pronounced marginal staining; major intervention necessary for improvement	2.4.1 Intense hypersensitivity 2.4.2 Delayed with minor subjective symptoms 2.4.3 No clinical detectable sensitivity. Intervention necessary but not replacement	3.4 Caries with cavitation and suspected undermined caries localized and accessible can be repaired
5. Clinically poor (replacement necessary)	1.5 Very rough, unacceptable plaque retentive surface	2.5 Intense, acute pulpitis or non-vital tooth. Endodontic treatment is necessary, and restoration has to be replaced	3.5 Deep secondary caries or exposed dentin that is not accessible for repair

### Statistical analysis

Data were analyzed using IBM SPSS software package version 23 (IBM Corp., Armonk, NY, USA). The qualitative data was presented using percentages and numbers. Chi-square test and Monte Carlo correction were employed for comparison among various groups. To assess the variations in each study group at each time interval and the variations between time intervals, Friedman’s test was utilized. The significance level for all tests was established at 0.05.

## Results

The participant group included 12 patients, resulting in 36 restorations in total having a 100% recall rate. Assessment of restorations was performed at baseline (one week), three, six and twelve months. Clinical findings of the investigated groups were provided in Table 2.

**Table 2:** Comparison between the different studied periods according to postoperative sensitivity and marginal staining

		Baseline		3 months		6 months		12 months		*p*
		No	%	No	%	No	%	No	%	
Postoperative sensitivity	Group I									
	1	8	66.7	10	83.3	8	66.7	8	66.7	0.340
	2	4	33.3	2	16.7	4	33.3	3	25.0	
	3	0	0.0	0	0.0	0	0.0	1	8.3	
	Group II									
	1	9	75.0	11	91.7	11	91.7	10	83.3	0.585
	2	3	25.0	1	8.3	1	8.3	2	16.7	
	3	0	0.0	0	0.0	0	0.0	0	0.0	
	Group III									
	1	9	75.0	11	91.7	10	83.3	10	83.3	0.494
	2	3	25.0	1	8.3	2	16.7	2	16.7	
	3	0	0.0	0	0.0	0	0.0	0	0.0	
Marginal staining	Group I									
	1	12	100.0	12	100.0	10	83.3	9	75.0	0.061
	2	0	0.0	0	0.0	2	16.7	3	25.0	
	Group II									
	1	12	100.0	12	100.0	12	100.0	10	83.3	0.112
	2	0	0.0	0	0.0	0	0.0	2	16.7	
	Group III									
	1	12	100.0	12	100.0	11	91.7	11	91.7	0.392
	2	0	0.0	0	0.0	1	8.3	1	8.3	

*Fr* Friedman test, *p* p value for comparison between the studied categories

Regarding postoperative sensitivity Group I (Cention N without adhesive) exhibited slightly inferior results compared to the other groups at the 12-month evaluation with one case evolving from minor hypersensitivity (score2) to moderate (score3) hypersensitivity at the 12-month period. At the end of the 12-month, 66.7% recorded as score 1, 25% recorded as score 2 and 8.3% recorded as score 3. Group II (CentionN with an adhesive) and Group III (Filtek bulk fill) had similar initial sensitivity levels, which were transient and subsided in the following follow-up periods. Both revealed decreasing trends in score 2 over the 12-month evaluation period from 25 to 16% and yielded an ideal (score1) of 83.3%. There was no discernible statistical difference between the two groups (*p* = 0.494). For the three study groups, only clinically acceptable scores were available (scoring 1–3) at each assessment interval with no statistically significant difference between the groups (*p* < 0.05), and no treatment was required.

Regarding marginal staining none of the study groups represented any marginal staining up to the 3-month period (score 1,100%). In Group I (Cention N without adhesive), two volunteers developed minor (score 2) marginal staining at 6- months, which continued to the 12-month evaluation. Another participant showed minor staining in the 12-month period. The percentages for scores 1 and 2 were provided: 83.3% and 16.7% at 6- months, and 75% and 25% at 12- months, respectively. In Group II (Cention N with adhesive), two participants exhibited minor (score 2) marginal staining during the 12-month period, easily removed by polishing, with percentages of 83.3% for score 1 and 16.7% for score 2. Group III (Filtek bulk fill) had one volunteer with minor (score 2) marginal staining at 6 months which continued to the 12-months period, with percentages of 91.7% for score 1 and 8.3% for score 2. Despite the staining, the results are considered clinically acceptable with slightly inferior results in Group I in comparison to Group II and Group III (*p* = 0.061, *p* = 0.112), respectively. The three groups did not differ in a way that was statistically significant (*p* < 0.05), and only polishing is required.

Regarding recurrent caries none of the restored teeth in any study group displayed any signs of secondary caries over the evaluation period of a year.

## Discussion

The objective of this study was to evaluate the clinical performance of a bioactive alkasite-based restorative material, both with and without adhesive, in comparison to a bulk-fill composite in Class I cavities, which were selected due to their high C-factor. The clinical performance of the tested materials was assessed based on marginal staining, postoperative sensitivity, and secondary caries, utilizing the FDI criteria. The FDI criteria are considered more sensitive and discriminatory than the USPHS criteria for detecting early minor changes in short-term clinical trials [34]. Only relevant criteria for assessing the clinical performance of bioactive restoratives, as established in previous research, were employed [35]. In vitro studies contribute to the enhancement and preliminary evaluation of restorative materials. However, despite attempts to simulate clinical conditions, they do not fully reflect the clinical performance of materials due to the variability of oral environment parameters. Therefore, to effectively compare different restorative materials and assess the clinical performance of newly developed materials, well-designed randomized controlled clinical trials are essential. This approach has been adopted in the present study [36].

Resin composites have undergone significant advancements in formulation to overcome various clinical challenges [37, 38]. Innovations such as bulk placement techniques, simplified adhesion protocols, and novel filler technologies have enhanced their applicability. However, clinical concerns such as the lack of antibacterial properties, polymerization shrinkage, and technique sensitivity remain. Additionally, bulk fractures and secondary caries continue to be the leading causes of composite failure [39, 40]. To mitigate these issues, hybrid materials that combine the advantages of composites and glass ionomers have been developed. These include resin-modified glass ionomer cements, compomers, giomers, and, more recently, bioactive composites [41]. The bioactive material used in this trial was Cention N, classified as an “alkasite” material, a subgroup of composite resins. This innovative category incorporates alkaline fillers that become highly reactive in acidic environments, enabling the release of acid-neutralizing ions. In addition to alkasite fillers, Cention N contains silanized reactive FAS fillers (calcium-barium-aluminum-fluorosilicate glass) and silanized non-reactive fillers. It also features a unique iso-filler that minimizes polymerization shrinkage by acting as a spring, gradually expanding as the forces between fillers increase during polymerization. Due to its dual-curing properties, Cention N can be used as a bulk, full-volume replacement, making it a competitive alternative to bulk-fill resin composites, which offer the advantage of simplified application in fewer increments and reduced chairside time [13].

In this study, postoperative sensitivity was reported more frequently with Cention N when used without adhesive, whereas Cention N with adhesive and Filtek Bulk Fill exhibited clinically comparable outcomes. Our findings align with those of Hirani et al. [25], who reported higher postoperative sensitivity in Cention N restorations without adhesive compared to Activa^™^ and Equia Forte, with Equia Forte exhibiting the least sensitivity at the 24 h mark. They attributed the increased postoperative hypersensitivity in Cention N to its low volumetric shrinkage, resulting from the organic monomer component in its liquid phase. Similarly, Mushtaq et al. [16] compared the postoperative sensitivity of Cention N without adhesive to other materials, including GC Fuji IX and nano-hybrid composites. They found that while Cention N initially exhibited moderate postoperative sensitivity, its clinical performance improved over time. Previous clinical studies have also reported initial hypersensitivity in the days or weeks following the operative procedure, which subsequently resolved [32]. The transient nature of postoperative sensitivity observed at baseline, but resolving before subsequent evaluations, may be attributed to procedural factors such as caries excavation, rubber dam application, and tooth drying rather than the specific restorative material used [42].

Cention N with adhesive application demonstrated lower postoperative sensitivity, consistent with the findings of Gordan and Mjör [43], who observed that self-etch adhesives resulted in reduced postoperative sensitivity. They evaluated the postoperative sensitivity of posterior restorations using a resin-based restorative material with a self-etching primer and attributed the reduced sensitivity to the formation of an acid-resistant resin-dentin interdiffusion zone. This zone provides a strong seal, compensates for polymerization stress, and minimizes postoperative sensitivity [43]. Furthermore, the Tetric N-Bond Universal adhesive used in this trial is a mild-etching adhesive with a low concentration of acidic monomers, which helps establish an optimal seal and prevents microleakage [44]. Although limited in vivo studies have evaluated postoperative sensitivity in Cention N with and without adhesive, in vitro research has linked microleakage to sensitivity, staining, and secondary caries [1]. Studies by Sujith et al. [15], Meshram et al. [19], and Kini et al. [20] and Recen et al. [45], support our findings, showing that Cention N with adhesive exhibits lower microleakage than Cention N without adhesive or other restorative materials, consistent with our results. The reduced microleakage observed in Cention N may be attributed to the presence of an effective self-cure initiator combined with cross-linking methacrylate monomers, leading to a high degree of polymerization throughout the restoration’s depth. Additionally, Cention N contains iso-fillers that act as stress relievers, minimizing shrinkage forces and resulting in reduced microleakage and low volumetric shrinkage [18, 23]. Contrary to these findings, George et al. [46] reported that Cention N restorations could be placed with or without an adhesive and observed reduced microleakage in Cention N without adhesive.

Moreover, both materials exhibited minimal discoloration at clinically acceptable levels, which can be attributed to their low polymerization shrinkage characteristics. The adaptation of a restorative material at the margins is influenced by the forces generated during polymerization shrinkage, which can lead to debonding at the tooth-restoration interface and frequently result in marginal discoloration [47]. This minimal discoloration in Cention N may be due to its UDMA-based formulation and the presence of iso-fillers with a low modulus of elasticity, which help reduce stress and minimize polymerization shrinkage. Similarly, Filtek Bulk Fill contains aromatic urethane dimethacrylate (AUDMA) and a fragmentation monomer (AFM), both of which function as stress modulators to further reduce shrinkage [48]. Additionally, the bulk-fill placement technique used with both materials contributes to lower polymerization shrinkage. These findings are consistent with the study by Sharma et al. [49].

Marginal adaptation loss may also result from localized bond failure. While phosphoric acid etching enhances enamel bond strength, it can reduce dentin bond strength, potentially leading to adhesion failure. Therefore, as recommended by Van Landuyt et al. [50], selective etching was performed in Groups II and III in this study. Yao et al. [51] highlighted that applying an adhesive act as a C-factor compensator, with the adhesive layer serving as an intermediary stress reliever. The slight marginal discoloration observed in Group I may be attributed to the absence of enamel etching, which could have affected the marginal seal. This aligns with the findings of the 2-year follow-up study by Albelasy et al. [52], which compared the clinical performance of Cention N and Surefil One to that of a conventional bulk-fill resin composite (PowerFil) in Class I and/or Class II cavities. The study reported that Cention N without adhesive exhibited significantly reduced marginal integrity compared to the resin composite at the 2-year evaluation. However, no recurrent caries were detected in any of the restorations. Another relevant study by Oz et al. [53] evaluated the performance of Cention N without adhesive in Class II cavities over a 12-month period and found that, after one year, the alkasite-based restorative material showed comparable Bravo scores to resin composite for marginal discoloration.

In this clinical trial, none of the study groups exhibited recurrent caries. This outcome may be attributed to the standardized study parameters, and the inclusion of patients with good oral hygiene, or it could be due to the relatively short evaluation period. This finding is consistent with studies by Albelasy et al. [52] and Oz et al. [53], which reported no recurrent caries in Cention N restorations during their respective follow-up periods. Although some marginal deterioration was observed in restorations without adhesive, the absence of recurrent caries underscores the caries-preventive potential of Cention N, likely due to its alkaline properties and fluoride release. Several in vitro studies [18, 53, 54] have documented its ion release capacity in laboratory settings. Di Lauro et al. [10] demonstrated that Cention N effectively releases Ca^2^⁺ and F⁻ ions in response to changes in pH and over time. Furthermore, Vidal et al. [55] reported that Cention N exhibited the highest fluoride release among tested materials. Additionally, Francois et al. [41] suggested that Cention N’s chemistry closely resembles that of ion-releasing composites (IRCs), supporting its classification as a new material family due to its proven bioactivity, particularly when applied without dental adhesive.

A split-mouth design was employed in this study, which is considered particularly advantageous for the clinical evaluation of restorative materials, as patient-related factors such as diet, habits, and oral hygiene are consistent across all groups. It is important to note that there was no loss of follow-up or discontinuation of intervention among the recruited subjects, which increases the validity of the results. However, it is essential to acknowledge some limitations of the trial. For instance, some clinical professionals may perceive the manual mixing of Cention N restorations as a drawback. Additionally, future studies should focus on patients with moderate to high caries, where ion-releasing materials are crucial for aiding the remineralization process due to recurrent pH declines. To further enhance our understanding of innovative alkasite restorative materials, future research should consider extending the evaluation period and increasing the sample size.

## Conclusion

Both the alkasite-based restorative material and resin composite demonstrated clinically acceptable and comparable results after one year. However, Cention N without adhesive showed slightly inferior clinical results but equally acceptable.

## Data Availability

The datasets used and analyzed during the current study are available from the corresponding author on reasonable request.
